# Digital Health Literacy in the Training of Informal Caregivers – Community Intervention: Andreia Costa

**DOI:** 10.1093/eurpub/ckac131.338

**Published:** 2022-10-25

**Authors:** D Águas, M Paço, A Henriques, M Arriaga, A Costa

**Affiliations:** 1 USF, ACES Lisboa Norte, Lisbon, Portugal; 2 Centro de Investigação, Inovação e Desenvolvimento em Enfermagem de Lisboa, Escola Superior de Enfermagem de Lisboa, Lisbon, Portugal; 3 Instituto de Saúde Ambiental, Faculdade de Medicina da Universidade de Lisboa, Lisbon, Portugal; 4 DGS, Direção-Geral da Saúde, Lisbon, Portugal; 5 Católica Research Centre for Psychological, Family and Social Wellbeing, Universidade Católica Portuguesa, Lisbon, Portugal; 6 ESEL, Escola Superior de Enfermagem de Lisboa, Lisbon, Portugal

## Abstract

**Background:**

Health Literacy allows optimizing healthy lifestyles and preventive and health protective behaviors (DGS, 2019). Low literacy can lead to a greater number of hospitalizations, a more frequent use of emergency services and a lower prevalence of preventive attitudes in the field of health. Internet-based interventions could have a positive impact on informal caregivers, reducing the geographical barrier, promoting self-efficacy in managing their own emotions, reducing burden.

**Aim:**

Contribute to the training of informal caregivers of a primary health care unit in Lisbon, through the promotion of digital health literacy.

**Methods:**

The Community Intervention project was carried out in the context of a home visit, focusing on 11 informal caregivers, through the presentation of an interactive digital manual. It was based on the methodology of health planning, through the elaboration of a diagnosis of the situation, definition of priorities, setting of objectives, selection of strategies, operational preparation and evaluation (Imperatori & Giraldes, 1993).

**Results:**

It was found that not all informal caregivers have access to the internet or digital technologies, and it was necessary to deliver the printed manual. Caregivers who accessed the interactive digital manual rated its content as very important, having accessed the suggested links without difficulty. The possibility of forwarding the digital manual to other caregivers was valid for all.

**Conclusions:**

Digital technologies promote communication in terms of health promotion, contributing to universal access and digital training in health, giving individuals the opportunity to increase care for their own health. The creation of digital health tools must be directed to the characteristics of the population. For individuals with low digital literacy, simple technologies must be created and for those who cannot or do not want to use digital tools, adequate alternatives must be created.

**Key messages:**

• Primary care health professionals may use digital technologies to promote health literacy.

• Vulnerable groups with low digital health literacy need support to increase access to digital technology that can promote health literacy.

